# Supplementary figures and images for: The environmental risk assessment of cell-processing facilities for cell therapy in a Japanese academic institution
Source: PLoS One. 2020 Aug 5;15(8):e0236600. doi: 10.1371/journal.pone.0236600 (PMC7406055; doi:10.1371/journal.pone.0236600)

S1 Figure

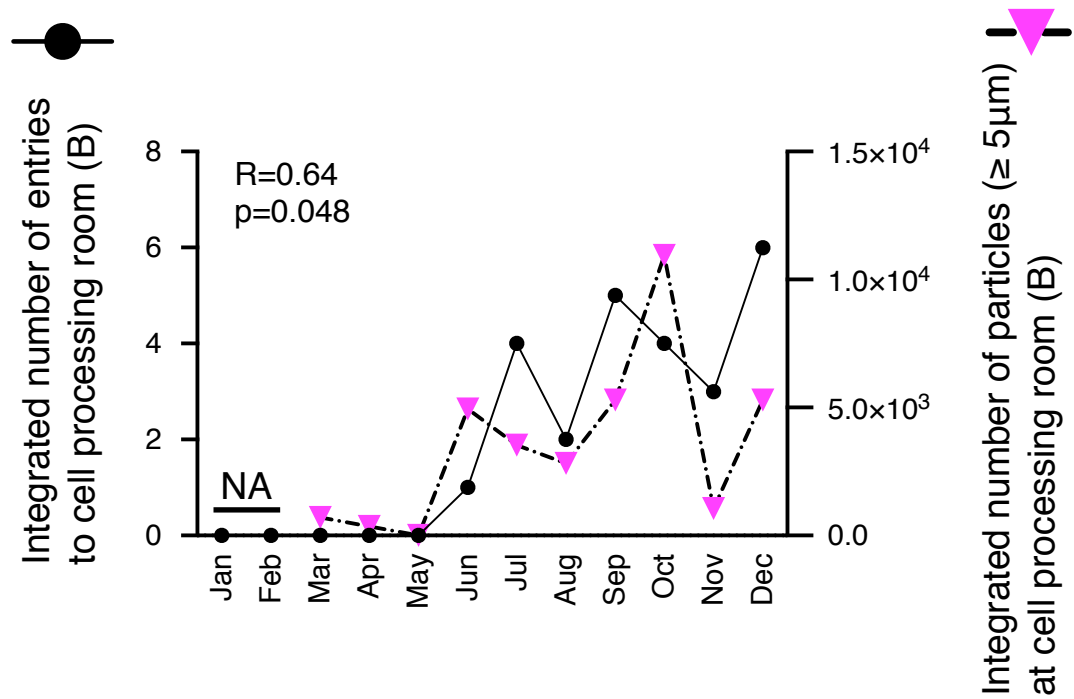

Supplement: S1 Fig — p = 0.048 by Pearson’s correlation coefficient (R = 0.64). NA: not assigned. (PDF) [file pone.0236600.s001.pdf]

S2 Figure

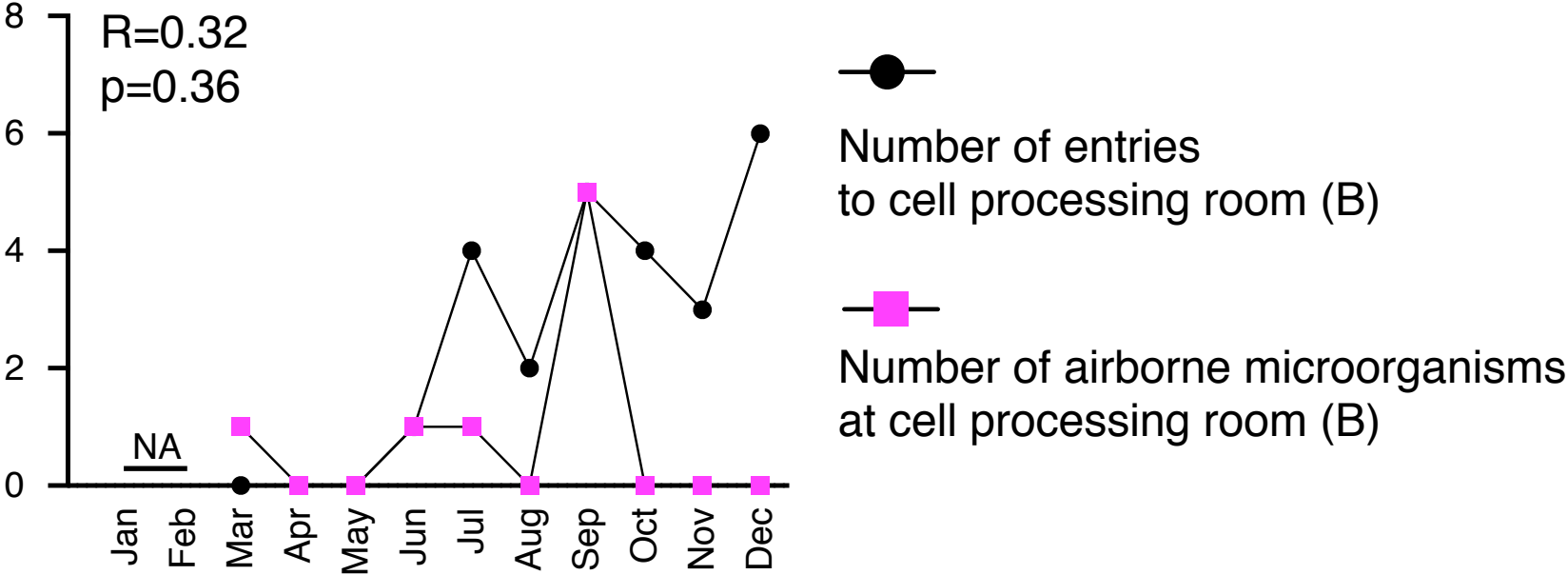

Supplement: S2 Fig — (PDF) [file pone.0236600.s002.pdf]

S3 Figure

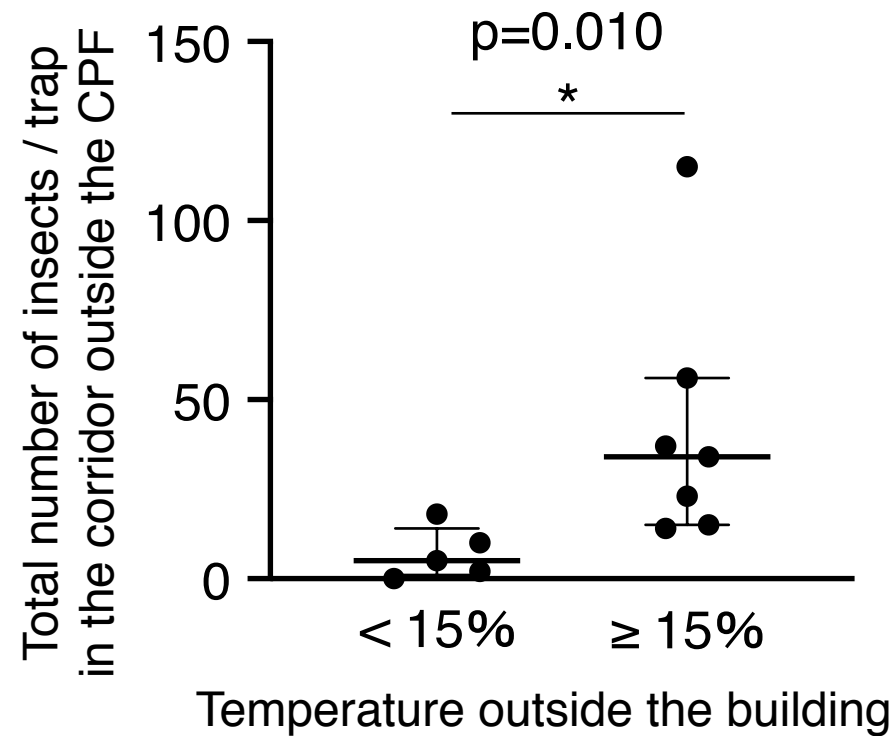

Supplement: S3 Fig — Data are shown by median with interquartile range. p values were calculated by the Mann Whitney U-test. (PDF) [file pone.0236600.s003.pdf]
