# Supplementary material for: The environmental risk assessment of cell-processing facilities for cell therapy in a Japanese academic institution
Source: PLoS One. 2020 Aug 5;15(8):e0236600. doi: 10.1371/journal.pone.0236600 (PMC7406055; doi:10.1371/journal.pone.0236600)
Supplement: S1 Table — (PDF) [file pone.0236600.s004.pdf]

S1 Table. Outline of the environmental definition by the Japanese Society for Regenerative Medicine (JSRM).

Cleanliness	Particles ≥ 0.5µm (at rest)	Microorganisms			
		Air sample	Settle plates	Contact plates	Glove print
	Number/m^3	CFU/m^3	CFU/plate (90mm)	CFU/plate (55mm)	CFU/glove (5 fingers)
A	3,520	<1	<1	<1	<1
B	3,520	10	5	5	5
C	352,000	100	50	25	-
D	3,520,000	200	100	50	-
