# Supplementary material for: The environmental risk assessment of cell-processing facilities for cell therapy in a Japanese academic institution
Source: PLoS One. 2020 Aug 5;15(8):e0236600. doi: 10.1371/journal.pone.0236600 (PMC7406055; doi:10.1371/journal.pone.0236600)
Supplement: S2 Table — (PDF) [file pone.0236600.s005.pdf]

Supplementary table 2. Sequence data of identified bacterium.

(I) Ralstonia pickettii strain 1F 16S ribosomal RNA gene, partial sequence
ATGATCTAGCTTGCTAGATTGATGGCGAGTGGCGAACGGGTGAGTAA
TACATCGGAACGTGCCCTGTAGTGGGGGATAACTAGTCGAAAGATTA
GCTAATACCGCATACGACCTGAGGGTGAAAGTGGGGGACCGCAAGGC
CTCATGCTATAGGAGCGGCCGATGTCTGATTAGCTAGTTGGTGAGGT
AAAGGCTCACCAAGGCGACGATCAGTAGCTGGTCTGAGAGGACGATC
AGCCACACTGGGACTGAGACACGGCCCAGACTCCTACGGGAGGCAGC
AGTGGGGAATTTTGGACAATGGGCGAAAGCCTGATCCAGCAATGCCG
CGTGTGTGAAGAAGGCCTTCGGGTTGTAAAGCACTTTTGTCCGGAAA
GAAATGGCTCTGGTTAATACCTGGGGTCGATGACGGTACCGGAAGAA
TAAGGACCGGCTAACTACGTGCCAGCAGCCGCGGTAATACGTAGGGT
CCAA

(II) Burkholderia lata strain CAB13001 16S ribosomal RNA gene, partial sequence
GCAGTCGAACGGCAGCACGGGTGCTTGCACCTGGTGGCGAGTGGCG
AACGGGTGAGTAATACATCGGAACATGTCCTGTAGTGGGGGATAGCC
CGGCGAAAGCCGGATTAATACCGCATACGATCTACGGATGAAAGCGG
GGGACCTTCGGGCCTCGCGCTATAGGGTTGGCCGATGGCTGATTAG
CTAGTTGGTGGGGTAAAGGCCTACCAAGGCGACGATCAGTAGCTGGT
CTGAGAGGACGACCAGCCACACTGGGACTGAGACACGGCCCAGACTC
CTACGGGAGGCAGCAGTGGGGAATTTTGGACAATGGGCGAAAGCCT
GATCCAGCAATGCCGCGTGTGTGAAGAAGGCCTTCGGGTTGTAAAGC
ACTTTTGTCCGGAAAGAAATCCTTGGCTCTAATACAGTCGGGGGATG
ACGGTACCGGAAGAATAAGCACCGGCTAACTACGTGCCAGCAGCCGC
GGTAATACGTAGGGTGCGAGCGTTAATCGGAATCACTGGGCGTAAAG
A

(III) Moraxella osloensis strain CFP312 16S ribosomal RNA gene, partial sequence

GGAAGCTGCCTTCGCCATCGGTATTCCTCCAGATCTCTACGCATTTC
ACCGCTACACCTGGAATTCTACTTCCCTCTCACATATTCTAGCACCAC
CAGTATCACATGCAGTTCCCAGGTTAAGCCCGGGGATTTCACATGTG
ACTTAATGAGCCACCTACACTCGCTTTACGCCCAGTAATTCCGATTAA
CGCTCGCACCCTCTGTATTACCGCGGCTGCTGGCACAGAGTTAGCCG
GTGCTTATTCTGCAGGTAACGTCTAATCTAATGGGTATTAACCATTA
GCCTCTCCTCCCTGCTTAAAGTGCTTTACAACCAAAAGGCCTTCTTCA
CACACGCGGCATGGCTGGATCAGGGTTGCCCCCATTGTCCAATATTC
CCC

(IV) Staphylococcus hominis partial 16S rRNA gene, isolate R5_C3_IIIB
GGAGCTTGCTCCTTTGACGTTAGCGGCGGACGGGTGAGTAACACGTA
GGTAACCTACCTATAAGACTGGGATAACTTCGGGAAACCGGAGCTAA
TACCGGATAATATTTCGAACCGCATGGTTCGATAGTGAAAGATGGCT
TTGCTATCACTTATAGATGGACCTGCGCCGTATTAGCTAGTTGGTAA
GGTAACGGCTTACCAAGGCAACGATACGTAGCCGACCTGAGAGGGTG
ATCGGCCACACTGGAACTGAGACACGGTCCAGACTCCTACGGGAGGC
AGCAGTAGGGAATCTTCCGCAATGGGCGAAAGCCTGACGGAGCAACG
CCGCGTGAGTGATGAAGGTCTTCGGATCGTAAAACTCTGTTATTAGG
GAAGAACAAACGTGTAAGTAACTGTGCACGTCTTGACGGTACCTAAT
CAGAAAGCCACGGCTAACTACGTGCCAGCAGCCGCGGTAATACGTAG
GTGGCAAGCGTTATCCGGAATC

(V) Sphingomonas paucimobilis strain E8T4224 16S ribosomal RNA gene, partial sequence
GCCGCGTGAGTGATGAAGGCCCTAGGGTTGTAAAGCTCTTTTACCCG
GGAAGATAATGACTGTACCGGGAGAATAAGCCCCGGCTAACTCCGTG
CCAGCAGCCGCGGTAATACGGAGGGGGCTAGCGTTGTTCGGAATTAC
TGGGCGTAAAGCGCACGTAGGCGGCTTTGTAAGTCAGAGGTGAAAGC
CTGGAGCTCAACTCCAGAACTGCCTTTGAGACTGCATCGCTTGAATC
CAGGAGAGGTCAGTGGAATTCCGAGTGTAGAGGTGAAATTCGTAGAT
ATTCGGAAGAACACCAGTGGCGAAGGCGGCTGACTGGACTGGTATTG

ACGCTGAGGTGCGAAAGCGTGGGGAGCAAACAGGATTAGATACCCNG
GTAG

(VI) Micrococcus yunnanensis partial 16S rRNA gene, isolate KMP789-MA53
GTGAGTAACACGTGAGTAACCTGCCCTTAACTCTGGGATAAGCCTGG
GAAACTGGGTCTAATACCGGATAGGAGCGTCCACCGCATGGTGGGTG
TTGGAAAGATTTATCGGTTTTGGATGGACTCGCGGCCTATCAGCTTG
TTGGTGAGGTAATGGCTCACCAAGGCGACGACGGGTAGCCGGCCTG
AGAGGGTGACCGGCCACACTGGGACTGAGACACGGCCCAGACTCCTA
CGGGAGGCAGCAGTGGGGAATATTGCACAATGGGCGCAAGCCTGAT
GCAGCGACGCCGCGTGAGGGATGACGGCCTTCGGGTTGTAAACCTCT
TTCAGTAGGGAAGAAGCGAAAGTGACGGTACCTGCAGAAGAAGCACC
GGCTAAC

(VII) Methylobacterium suomiense gene for 16S ribosomal RNA, partial sequence, strain: 92a-12
CGGCAGACGGGTGAGTAACACGTGGGAACGTGCCCTTCGGTTCGGA
ATAACTCAGGGAAACTTGAGCTAATACCGGATACGCCCTTTTGGGGA
AAGGTTTACCGCCGAAGGATCGGCCCGCGTCTGATTAGCTAGTTGGT
GGGGTAACGGCCTACCAAGGCGACGATCAGTAGCTGGTCTGAGAGGA
TGATCAGCCACACTGGGACTGAGACACGGCCCAGACTCCTACGGGAG
GCAGCAGTGGGGAATATTGGACAATGGGCGCAAGCCTGATCCAGCCA
TGCCGCGTGAGTGATGAAGGCCTTAGGGTTGTAAAGCTCTTTTGTCC
GGGACGATAATGACGGTACCGGAAGAATAAGCCCCGGCTAACTTCGT
GCCAGCAGCCGCGGTAATACGAAGGGGGCTAGCGTTGCTCGGAATCA
CTGGGCGTAAAGA

(VIII) Cladosporium cladosporioides strain CBS 129108 large subunit ribosomal RNA gene, partial sequence
TAGCTTTAGATGAAATTTACCACCCATTTAGAGCTGCATTCCCAAACA
ACTCGACTCGTCGAAGGAGCTACGTATAGAGCACCTTTCCGACCGCA
TACGGGATTCTCACCCTCTATGACGTCCTGTTCCAAGGAACTTAGGT

CGGTGGTTACTCAGAAGCATCCTCTACAAATTACAACTCGGACGTCG
AAGACGCCAGATTTCAAATTTGAGCTATTGCTGCTTCACTCGCCGTTA
CTAGAGCAATCCCTGTTGGTTTCTTTTCCTCCGCTTATTGATATGC

(IX) Irpex lacteus strain ZJLD 002 internal transcribed spacer 1, partial sequence; 5.8S ribosomal RNA gene and internal transcribed spacer 2, complete sequence; and large subunit ribosomal RNA gene, partial sequence
AGCTGGCCTTTCCGAGGCATGTGCACGCCTGGCTCATCCACTCTTAA
CCTCTGTGCACTTTATGTAAGAGAAAAAAATGGTGGAAGCTTCCAGG
ATCTCGCGAGAGGTCTTCGGTTGAACAAGCCGTTTTTCTTTCTTATG
TTTTACTACAAACGCTTCAGTTATAGAATGTCAACTGTGTATAACACA
TTTATATACAACTTTCAGCAACGGATCTCTTGGCTCTCGCATCGATGA
AGAACGCAGCGAAATGCGATAAGTAATGTGAATTGCAGAATTCAGTG
AATCATCGAATCTTTGAACGCACCTTGCACTCCTTGGTATTCCGAGGA
GTATGCCTGTTTGAGTCTCATGGTATTCTCAACCCCTAAATTTTTGTA
ATGAAGGTTTAGCGGGCTTGGACTTGGAGGTTGTGTCGGCCCTTGT
CGGTCGACTCCTCTGAAATGCATTAGCGTGAATCTTACGGATCGCCT
TCAGTGTGATAATTATCTGCGCTGTGGTGTTGAAGTATTTATGGTGT
TCATGCTTCGAACCGTCTCCTTGCCGAGACAATCATTTGACAATCTGA
GCTCAAATCAGGTAGGACTACCCGCTGAACTTAAGCATAT
