# Supplementary material for: The environmental risk assessment of cell-processing facilities for cell therapy in a Japanese academic institution
Source: PLoS One. 2020 Aug 5;15(8):e0236600. doi: 10.1371/journal.pone.0236600 (PMC7406055; doi:10.1371/journal.pone.0236600)
Supplement: S3 Table — (PDF) [file pone.0236600.s006.pdf]

S3 Table. Sterility in cell processing products.

Product No.	Sterility test
01	Negative
26	Negative
13	Negative
04	Negative
07	Negative
15	Negative
